# CT-derived fractional flow reserve (FFRct) for functional coronary artery evaluation in the follow-up of patients after heart transplantation

**DOI:** 10.1007/s00330-021-08246-5

**Published:** 2021-09-15

**Authors:** Ricardo P. J. Budde, Fay M. A. Nous, Stefan Roest, Alina A. Constantinescu, Koen Nieman, Jasper J. Brugts, Lynne M. Koweek, Alexander Hirsch, Jonathon Leipsic, Olivier C. Manintveld

**Affiliations:** 1 Department of Radiology and Nuclear Medicine, Erasmus MC, University Medical Center Rotterdam, Doctor Molewaterplein 40, 3015 GD Rotterdam, The Netherlands; 2 Department of Cardiology, Thorax Center, Erasmus MC, University Medical Center Rotterdam, Doctor Molewaterplein 40, 3015 GD Rotterdam, The Netherlands; 3 Department of Radiology, Duke University Medical Center, 10 Duke Medicine Cir, Durham, NC 27710-1000 USA; 4 Department of Radiology, Providence Health Care, St. Paul’s Hospital, University of British Columbia, 1081 Burrard St, Vancouver, BC V6Z1Y6 Canada

**Keywords:** Computed tomography angiography, Coronary stenosis, Coronary vessels, Heart transplantation, Fractional flow reserve myocardial

## Abstract

**Objectives:**

Invasively measured fractional flow reserve (FFR) is associated with outcome in heart transplant (HTx) patients. Coronary computed tomography angiography (CCTA)–derived FFR (FFRct) provides additional functional information from anatomical CT images. We describe the first use of FFRct in HTx patients.

**Methods:**

HTx patients underwent CCTA with FFRct to screen for cardiac allograft vasculopathy. FFRct was measured distal to each coronary stenosis > 30% and FFRct ≤ 0.8 indicated hemodynamically significant stenosis. FFRct was also measured at the most distal location of each vessel. Overall distal FFRct was calculated as the mean of the distal values in the left, right, and circumflex coronary artery in each patient.

**Results:**

Seventy-three patients (age 56 (42–65) years, 63% males) at 11 (8–16) years after HTx were included. Eighteen (25%) patients had a focal hemodynamically significant stenosis (stenosis > 30% with FFRct ≤ 0.8). In the 55 patients without a hemodynamically significant focal FFRct stenosis (FFRct > 0.80), the distal left anterior descending artery FFRct was < 0.90 in 74% of the patients and 10 (18%) patients had ≥ 1 coronary artery with a distal FFRct ≤ 0.8, including 1 with a distal FFRct ≤ 0.8 in all coronaries. Overall distal FFRct in patients without focal stenosis was 0.88 (0.86–0.91), 0.87 (0.86–0.90), and 0.88 (0.86–0.91) (median with 25th–75th percentile) at 5–9, 10–14, or ≥ 15 years post-transplantation, respectively (*p* = 0.93).

**Conclusions:**

FFRct performed on CCTA scans of HTx patients demonstrated that 25% of patients had a focal coronary stenosis with FFRct ≤ 0.8. Even without a focal stenosis, FFRct values are often abnormal in HTx patients.

**Key Points:**

• *This is the first report describing the use of FFRct in in heart transplant patients.*

• *FFRct identifies patients after heart transplantation with hemodynamically significant coronary stenosis.*

• *Even without a focal stenosis, FFRct values are often abnormal in heart transplant patients.*

**Supplementary Information:**

The online version contains supplementary material available at 10.1007/s00330-021-08246-5.

## Introduction

Cardiac allograft vasculopathy (CAV) is an accelerated fibroproliferative disease that affects the coronary arteries in heart transplant (HTx) patients leading to coronary stenoses [1, 2]. Data from the International Society for Heart and Lung Transplantation (ISHLT) show that almost 50% of patients have CAV at 10 years post-transplant [3, 4]. CAV is the third to fourth leading cause of death amongst HTx patients and accounts for 1 in 8 deaths in those that survive the first year after HTx [3]. Medical treatment can slow CAV progression. But ultimately, CAV progresses and usually revascularization, and in select cases, even retransplantation is needed [1]. Patients with CAV seldom present with classical symptoms of angina because the transplanted heart is denervated [1]. The ISHLT currently recommends annual or biannual invasive coronary angiography (ICA) to evaluate for the development of CAV [5]. Beyond anatomical evaluation invasive fractional flow reserve (FFR) measurements have been shown to provide complementary information and has been shown to be an independent predictor of death and retransplantation [6].

Coronary computed tomography angiography (CCTA) is a reliable alternative to ICA for CAV detection [7]. Technology to calculate FFR values based on CCTA images (FFRct) has become commercially available and is validated in multiple studies in chest pain patients [8–10]. The use of FFRct in the follow-up of HTx patients has not been reported yet. The aim of this study is to describe the initial results of CCTA with FFRct analysis in a cohort of HTx patients.

## Materials and methods

### Patient selection

All HTx patients from one hospital that participated in the Assessing Diagnostic Value of Non-invasive FFRct in Coronary Care (ADVANCE EXTEND) registry were included [8]. The institutional ethics committee approved the study. All patients provided informed consent. Patients with a stent in the left main, a stent in two or more major coronary arteries or a metallic stent in the left coronary system could not be enrolled in the ADVANCE EXTEND registry as these scans cannot be processed for FFRct analysis. For each patient, we recorded the following: patient demographics, angina symptom status, current medication use, and clinical outcomes at 1 year including any additional test that was performed for coronary ischemia testing, coronary revascularization, and the occurrence of major adverse cardiac events (MACE) defined as myocardial infarction, all-cause mortality, or unplanned hospitalization for acute coronary syndrome leading to revascularization. The CAV score prior to the FFRct analysis was determined by combining all available information including findings at ICA and stress scintigraphy closest to the CCTA with FFRct.

In our hospital, patients undergo an annual CCTA starting the 5th year post-transplant for CAV surveillance. Invasive coronary angiography is routinely performed one and four years post-transplant and thereafter only when clinically indicated (e.g., stenosis detected on CCTA).

### CCTA acquisition

A contrast-enhanced CCTA examination was performed according to the normal clinical routine on a dual-source CT scanner (Force or Drive, Siemens Healthineers) using a prospectively ECG-triggered acquisition mode. Automated tube voltage and tube current selection were used. Contrast injection was generally done using a test-bolus injection with 10–15 ml of contrast media (iopromide 370 mg/ml, Bayer) followed by a 20-ml saline chaser. The CCTA was then performed using 50–70 ml of contrast material followed by a 25-ml saline chaser. Flow rate was 5.4 ml/s. Beta-blockers to lower the heart rate at the time of scanning were administered in conjunction with the treating cardiologist. Sublingual nitroglycerine was administered just prior to scanning in each patient.

### FFRct analysis

**Fig. 1:**
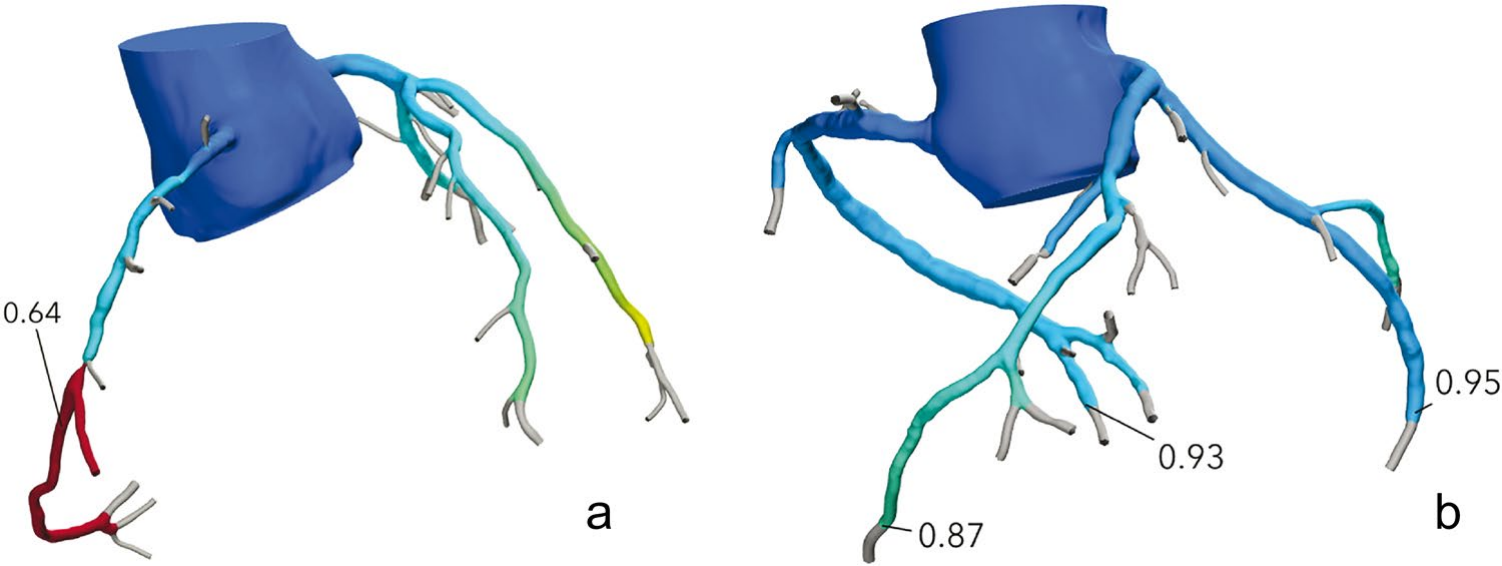
**a** CT-derived fractional flow reserve (FFRct) coronary tree with a focal stenosis in the right coronary artery (RCA) and FFRct value of 0.64; **b** FFRct coronary tree in a patient without a significant stenosis with distal FFR measurements in the tree major coronary arteries: RCA 0.93, left anterior descending (LAD) 0.87, and left circumflex (LCx) 0.95; with a mean distal FFRct of 0.92

All CCTA scans were sent to Heartflow for FFRct analysis. In brief, the analysis is based on defining the coronary artery boundaries, subsequently extracting a 3D model of the coronary arteries which is used to perform computational fluid dynamics calculations. Ultimately, a 3D coronary model is generated that can be interrogated at each point in a coronary artery to provide the FFR value at that specific location. In case a modeled stenosis, > 30% is present in a coronary artery; the FFR value distal to the stenosis is automatically supplied on the FFRct report and recorded [11]. A FFRct value of ≤ 0.8 was considered positive and to constitute a hemodynamically significant stenosis (Fig. 1a). Also, in each patient, the FFRct value at the most distal point in each of the three major coronary arteries (right coronary artery (RCA), left anterior descending (LAD), and left circumflex (LCx)) was recorded and the overall FFRct was calculated in each patient as the mean of the three FFRct values (Fig. 1b). A distal FFRct value of ≤ 0.8 without focal coronary stenosis was not considered hemodynamically significant stenosis. In the case of a total occlusion, a FFRct value of 0.5 was used for analysis similar to a previous study using FFRct [11]. The volume-to-mass ratio (V/M, volume of the coronary arteries divided by the left ventricular myocardial mass) was derived from the FFRct segmentation [12].

### Statistical analysis

Data are presented as absolute numbers, means with standard deviation or median with 25th–75th percentile where appropriate. Patient subgroups were compared using Mann–Whitney-*U*, Kruskal–Wallis, chi-square, or Fisher’s exact tests depending on the type of data. For analysis of time after HTx, patients were divided into three groups: up to 10 years, 10 to 15 years, and 15 or more years after HTx. A *p*-value of < 0.05 was considered significant.

## Results

### Patients

Seventy-three HTx patients (46 males (63%), age at time of CCTA 56 (42–65) years, age at HTx 43 (26–54) years) who were 11 (8–16) years after HTx were included (Table 1). The CAV score prior to CCTA was CAV 0 in 60 (82%) patients, CAV 1 in 3 (4%) patients, and CAV 2 in 10 (14%) patients. Fifteen (21%) patients were on mammalian target of rapamycin receptor inhibitor (mTORi). Sixty patients (82%) were on statins and/or ezetimibe. Beta-blockers were administered prior to the CT scan in 51 (70%) patients. The median heart rate of the patients included in the study was 74 (66–84) beats per minute.

**Table 1:** Baseline patient characteristics

Total number of patients, *n*		73
Age, years		56 (42–65)
Recipient gender, % male		46 (63%)
Race	Asian	2 (3%)
	Black or African American	1 (1%)
	White	70 (96%)
Ethnicity	Hispanic or Latino	1 (1%)
	Non-Hispanic or Latino	72 (99%)
Body mass index, kg/m^2^		26 (24–29)
CAV status prior to CCTA	0	60 (82%)
	1	3 (4%)
	2	10 (14%)
	3	0 (0%)
Angina status	Typical	0 (0%)
	Atypical	0 (0%)
	None	73 (100%)
Diabetes mellitus		22 (30%)
Insulin use		10 (14%)
Hypertension		61 (84%)
Smoking	Current	1 (1%)
	Previous	23 (32%)
	Never	49 (67%)
Blood creatinine, µmol/L		107 (86–126)
Left ventricular function < 50%		3 (4%)
Time since HTx, years		11 (8–16)
Reason for HTx	Ischemic heart disease	17 (23%)
	Other	56 (77%)
Recipient age at HTx, years		43 (26–54)
Donor age, years		39 (21–47)
Donor gender, % male		32 (44%)
Donor body mass index, kg/m^2^		23 (21–24)
Diabetes mellitus recipient prior to HTx		0 (0%)
CMV within first year post-HTx		12 (16%)
Total cellular-mediated rejection periods per patient		1 (0–2)
Total antibody-mediated rejection periods per patient		0 (0–0)
Patients with coronary stent in cardiac transplant		6 (8%)
Pacemaker present in cardiac transplant		17 (23%)
Statin and/or ezetimibe use		60 (82%)
Thrombocyte aggregation inhibitors and/or oral anticoagulant use		69 (95%)
Current immunosuppressive regimen	mTORi	15 (21%)
	CNI	73 (100%)
	Steroids	52 (71%)
	Mycophenolate mofetil	25 (34%)
	Purine antagonists	1 (1%)

*HTx*, heart transplantation; *CAV*, cardiac allograft vasculopathy; coronary computed tomography angiography; *CMV*, cytomegalovirus; *mTORi*, mammalian target of rapamycin receptor inhibitor (sirolimus, everolimus); *CNI*, calcineurin inhibitor (prograft, cyclosporin); steroids (prednisone); mycophenolate mofetil (cellcept); purine antagonists (azathioprine)

### FFRct analysis

FFRct could be calculated in all patients in 214 coronary arteries including one with a stent (image quality was very good in this vessel and the stent created almost no artifacts). The LAD could not be analyzed in 3 patients and the LCx in 2 patients due to the presence of metallic stents. Eighteen (25%) patients had ≥ 1 focal coronary stenosis of > 30% with a FFRct value of ≤ 0.8. In the 55/73 (75%) patients without a focal stenosis with FFRct ≤ 0.8, the distal LAD FFRct was available for 54 vessels of which 40 (74%) had a distal LAD FFRct value of < 0.90. Ten out of the 55 (18%) patients had ≥ 1 coronary artery with a distal FFRct ≤ 0.8, including 1 patient that had an overall distal FFRct of ≤ 0.8. The V/M was available in 70 patients and was 25.4 (21.5–35.8) for the entire cohort.

### Focal stenosis with FFRct ≤ 0.8 vs. no focal stenosis

Compared to those without a hemodynamically significant focal stenosis, patients with a focal stenosis and FFRct ≤ 0.8 had significantly longer time after HTx (15 (11–18) years vs. 10 (7–15) years, *p* = 0.02); had higher CAV scores prior to CCTA (*p* = 0.02); and more often had a coronary stent (*p* = 0.03) (Table 2). In patients with at least one focal stenosis > 30% with focal FFRct ≤ 0.8, the overall distal FFRct in the other coronary arteries (so only including those vessels without a focal > 30% stenosis with FFRct ≤ 0.8) was similar to patients that had no focal stenosis: 0.88 (0.86–0.91) vs. 0.88 (0.82–0.90), respectively (*p* = 0.15) (Table 3).

**Table 2:** Comparison of patient groups based on FFRct results

		Patients without focal stenosis with FFRct ≤ 0.8	Patients with focal FFRct ≤ 0.8	All patients	*p*-value
Number of patients, *n*		55	18	73	
V/M ratio		26.4 (21.6–35.8)	25.3 (18.9–27,7)	25.4 (21.5–35.8)	0.26
Time since transplantation, years		10 (7–15)	15 (11–18)	11 (8–16)	0.02
CAV status prior CCTA	CAV 0	49 (89%)	11 (63%)	60 (82%)	0.02
	CAV 1	1 (2%)	2 (11%)	3 (4%)	
	CAV 2	5 (9%)	5 (6%)	10 (14%)	
	CAV 3	0 (0%)	0 (0%)	0 (0%)	
Coronary stent present in cardiac transplant		2 (4%)	4 (22%)	6 (8%)	0.03
Current medication use	Statin and/or ezetimibe use	46 (84%)	14 (78%)	60 (82%)	0.72
	Thrombocyte aggregation inhibitors and/or oral coagulant use	52 (95%)	17 (94%)	69 (95%)	1.00
	Insulin use	6 (11%)	4 (22%)	10 (14%)	0.25
	mTORi	11 (20%)	4 (22%)	15 (21%)	1.00
	CNI	55 (100%)	18 (100%)	73 (100%)	-
	Steroids	36 (66%)	16 (89%)	52 (71%)	0.06
	Mycophenolate mofetil	20 (36%)	5 (28%)	25 (34%)	0.51
	Purine antagonists	1 (2%)	0 (0%)	1 (1%)	1.00
1-year follow-up					
Additional ischemia test performed	All tests combined	3 (6%)	10 (56%)	13 (18%)	< 0.001
	ICA	3 (5%)	10 (56%)	13 (18%)	
	Invasive FFR	2 (4%)	6 (33%)	8 (11%)	
	SPECT	0 (0%)	1 (6%)	1 (1%)	
	Other non-invasive imaging	0 (0%)	1 (6%)	1 (1%)	
Revascularization performed		1 (2%)	7 (39%)	8 (11%)	< 0.001
MACE		2 (4%)	1 (6%)	3 (4%)	1.00

*FFRct*, fractional flow reserve computed tomography; *CAV*, cardiac allograft vasculopathy; *CCTA*, coronary computed tomography angiography; *mTORi*, mammalian target of rapamycin receptor inhibitor (sirolimus, everolimus); *CNI*, calcineurin inhibitor (tacrolimus, cyclosporin); steroids (prednisone); mycophenolate mofetil (CellCept); purine antagonists (azathioprine); *ICA*, invasive coronary angiography; *SPECT*, single-photon emission computed tomography; *MACE*, major adverse cardiovascular event. Data are presented as absolute numbers with percentages or median with 25th–75th percentile where appropriate

**Table 3:** Comparison of FFRct in vessels without hemodynamically significant stenosis in patient groups with at least one hemodynamically significant stenosis on FFRct versus patient groups without any hemodynamically significant stenosis on FFRct

		Patients without hemodynamically significant stenosis on FFRct	Patients with hemodynamically significant stenosis on FFRct	*p*-value
Overall distal FFRct*		0.88 (0.86–0.91) *n* = 55	0.88 (0.82–0.90) *n* = 17	0.15
Distal FFRct per vessel*	RCA	0.90 (0.88–0.92) *n* = 55	0.88 (0.85–0.91) *n* = 13	0.09
	LAD	0.87 (0.82–0.90) *n* = 54	0.87 (0.81–0.87) *n* = 6	0.48
	LCx	0.90 (0.86–0.94) *n* = 55	0.88 (0.86–0.91) *n* = 8	0.25
Number of vessels with distal FFRct < 0.94*	RCA	50 (91%)	12 (92%)	1.00
	LAD	51 (94%)	6 (100%)	1.00
	LCx	39 (71%)	7 (88%)	0.43
Number of vessels with distal FFRct < 0.90*	RCA	24 (44%)	9 (69%)	0.10
	LAD	40 (74%)	6 (100%)	0.32
	LCx	24 (44%)	6 (75%)	0.14
Number of vessels with distal FFRct ≤ 0.80*	RCA	1 (2%)	2 (15%)	0.09
	LAD	9 (17%)	1 (17%)	1.00
	LCx	3 (6%)	1 (13%)	0.43

*FFRct*, fractional flow reserve computed tomography; *RCA*, right coronary artery; *LAD*, left anterior descending; *LCx*, left circumflex; *NA*, not applicable. *Vessel(s) were included in this group if they had a distal FFRct but no hemodynamically significant stenosis. Data are presented as absolute numbers or median with 25th–75th percentile where appropriate

### Time after HTx

Patients at a longer time since HTx more often had a coronary stenosis with FFRct ≤ 0.8 (Table 4). The overall distal FFRct as well as per vessel and the V/M did not differ significantly between groups. In the 55 patients without a focal stenosis with FFR ≤ 0.8, both the mean distal FFR and per vessel separately did not differ over time (Table 5).

**Table 4:** Comparison of patient groups based on time after HTx

		5–9 years post-HTx	10–14 years post-HTx	≥ 15 years post-t post-HTx	*p*-value
Number of patients		28	20	25	
CAV status prior CCTA	CAV 0	25 (89%)	16 (80%)	19 (76%)	0.74
	CAV 1	1 (4%)	1 (5%)	1 (4%)	
	CAV 2	2 (7%)	3 (15%)	5 (20%)	
	CAV 3	0 (0%)	0 (0%)	0 (0%)	
Coronary stent present		1 (4%)	2 (10%)	3 (12%)	0.51
Current medication use	Statin and/or ezetimibe use	25 (89%)	16 (80%)	19 (76%)	0.43
	Thrombocyte aggregation inhibitors and/or oral coagulant use	26 (93%)	19 (95%)	24 (96%)	0.88
	Insulin use	1 (4%)	4 (20%)	5 (20%)	0.14
	mTORI	5 (18%)	4 (20%)	6 (24%)	0.86
	CNI	28 (100%)	20 (100%)	25 (100%)	-
	Steroids	17 (61%)	15 (75%)	20 (80%)	0.27
	Mycophenolate mofetil	14 (50%)	6 (30%)	5 (20%)	0.064
	Purine antagonists	1 (4%)	0 (0%)	0 (0%)	0.44
Prevalence of patients with a focal stenosis (> 30%) with FFRct ≤ 0.8		2 (7%)	6 (30%)	10 (40%)	0.02
Overall distal FFRct		0.88 (0.86–0.91)	0.87 (0.83–0.90)	0.86 (0.79–0.90)	0.13
Distal FFRct per vessel	RCA	0.91 (0.88–0.92)	0.90 (0.86–0.93)	0.88 (0.85–0.91)	0.12
	LAD	0.87 (0.82–0.90)	0.87 (0.79–0.89)	0.84 (0.77–0.87)	0.077
	LCx	0.90 (0.85–0.93)	0.88 (0.85–0.94)	0.90 (0.86–0.94)	0.91
V/M		25.37 (19.83–29.76)	25.36 (21.93–27.82)	26.91 (21.58–37.68)	0.73
1 year follow-up					
Additional ischemia test performed	All tests combined	0 (0%)	3 (15%)	10 (40%)	0.001
	ICA	0 (0%)	3 (15%)	10 (40%)	
	Invasive FFR	0 (0%)	2 (10%)	6 (24%)	
	SPECT	0 (0%)	0 (0%)	1 (4%)	
	Other	0 (0%)	0 (0%)	1 (4%)	
Revascularization performed		0 (0%)	2 (10%)	6 (24%)	0.02
MACE		1 (4%)	0 (0%)	2 (8%)	0.40

*HTx*, heart transplantation; *FFRct*, fractional flow reserve computed tomography; *CAV*, cardiac allograft vasculopathy; *CCTA*, coronary computed tomography angiography; *mTORi*, mammalian target of rapamycin receptor inhibitor (sirolimus, everolimus); *CNI*, calcineurin inhibitor (tacrolimus, cyclosporin); steroids (prednisone); mycophenolate mofetil (CellCept); *ICA*, invasive coronary angiography; *SPECT*, single-photon emission computed tomography; *MACE*, major adverse cardiovascular event; *V/M*, volume-to-mass ratio. Data are presented as absolute numbers or median with 25th–75th percentile where appropriate

**Table 5:** Comparison of patient groups without focal stenosis based on time after transplantation

		5–9 years post-HTx	10–14 years post-HTx	≥ 15 years post-Htx	*p*-value
Number of patients		26	14	15	
Overall distal FFRct		0.88 (0.86–0.91)	0.87 (0.86–0.90)	0.88 (0.86–0.91)	0.93
Distal FFRct per vessel	RCA	0.91 (0.88–0.92)	0.90 (0.87–0.93)	0.89(0.87–0.91)	0.39
	LAD	0.87 (0.82–0.90)	0.87 (0.84–0.89)	0.84(0.81–0.88)	0.53
	LCx	0.90 (0.86–0.93)	0.89 (0.85–0.94)	0.91 (0.90–0.95)	0.19

*HTx*, heart transplantation; *FFRct*, fractional flow reserve computed tomography; *RCA*, right coronary artery; *LAD*, left anterior descending; *LCx*, left circumflex

### Follow-up

At 1-year follow-up, 13 (18%) patients had undergone an additional test to assess the coronary arteries including 13 ICA, of which 8 with invasive FFR measurements, 1 stress scintigraphy, and 1 cardiac magnetic resonance scan (Tables 2 and 4). Additional testing was performed more frequently in case of a focal stenosis with FFRct ≤ 0.8 (*p* < 0.001) and in patients at a longer time after HTx (*p* = 0.001).

**Fig. 2:**
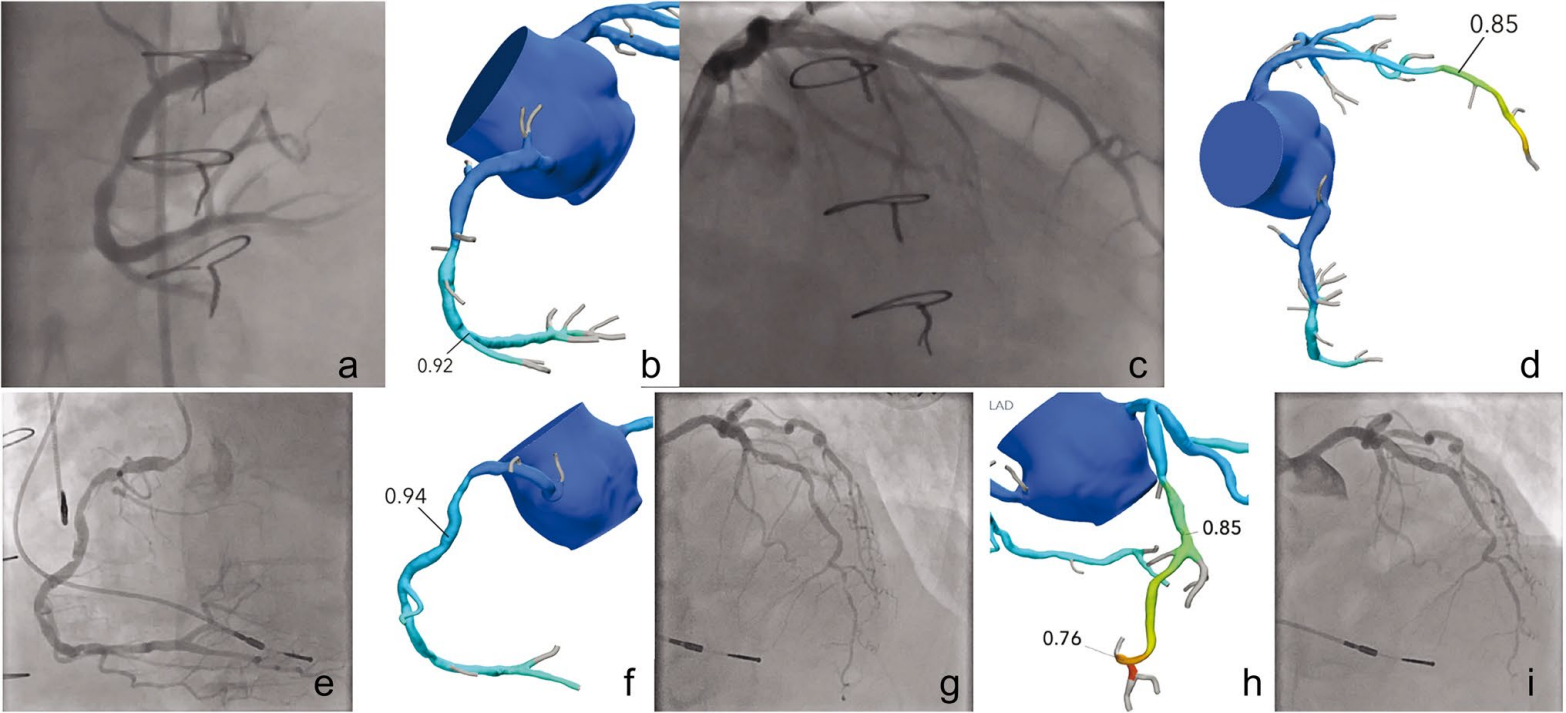
Patient with a focal stenosis in the right coronary artery (RCA) and mid left anterior descending (LAD) of > 30% with FFRct value of 0.92 and 0.85, respectively (**b**, **d**). Invasive coronary angiography (ICA) was performed that confirmed the FFRct finding of non-significant stenosis with an invasive FFR value of 0.96 in the RCA and 0.83 in the LAD (**a**, **c**). Patient with a focal stenosis in the RCA and LAD of > 30% with FFRct value of 0.94 and 0.76 respectively (**f**, **h**). ICA confirmed the lesion in the RCA to be non-significant (invasive FFR 0.95) and stenoses in the LAD (**e**, **g**). Additional stress scintigraphy demonstrated ischemia in the LAD territory and the LAD was stented in a subsequent session with good result (**i**)

Three patients that underwent additional testing did not have a focal stenosis with FFRct ≤ 0.8 (Fig. 2). The ICA was requested based on CCTA findings before FFRct results were reviewed. The FFRct and ICA results of all 13 patients are detailed in the electronic supplementary material. Three patients did not have a focal stenosis with FFRct < 0.8 but did undergo ICA and one received revascularization of the LAD (FFRct 0.83) (electronic supplementary material). In the 10 patients with a FFRct ≤ 0.8 who underwent ICA, 7 underwent revascularization as the coronary stenosis was demonstrated to be significant either by an invasive FFR measurement ≤ 0.8, visual interpretation of the ICA, or findings at optical coherence tomography during the same session (Fig. 2). Of the 3 patients with a FFRct ≤ 0.8 who underwent ICA but were not revascularized, one did show chronic total occlusions, but these could not be treated; the other 2 patients did not show significant stenoses.

Three patients had a MACE during follow-up, one being an iatrogenic infarction due to no reflow after stenting, one patient suffered a stroke after LAD stenting and later died of carcinoma, and one patient died of sudden cardiac death of unknown cause 10 months post-CCTA although the CCTA showed no abnormalities, and the patient had a coronary calcium score of 0.

## Discussion

We describe the first cohort of HTx patients that underwent FFRct analysis of CCTA performed for routine annual screening for CAV. In our analysis, patients with a coronary stenosis and FFRct ≤ 0.8 were at a longer time since transplantation and more often underwent additional testing for coronary ischemia and revascularization.

### Cardiac allograft vasculopathy

CAV constitutes a serious complication affecting HTx patients. The cause of CAV is likely multifactorial consisting of both alloimmune dependent and independent factors and has both donor and recipient related risk factors [13]. The ISHLT recommends (bi)annual ICA for screening but other techniques are used as well depending on local expertise and preferences including amongst others stress scintigraphy, intravascular ultrasound (IVUS), stress echocardiography, and CCTA [5, 14, 15]. Ideally, a technique would not only detect anatomical findings of CAV but also be able to predict its occurrence before structural changes become apparent. When medical treatment to slow down CAV progression should be initiated is a clinical dilemma, starting too early unnecessarily exposes the patient to side effects of the medication whereas starting too late appears to be ineffective due to a different plaque composition [1].

### FFR measurements in transplant patients

Several studies describe invasive FFR measurements in HTx patients [6, 16–18]. Fearon et al. described 53 patients *without angiographic disease* at a mean of 3.1 ± 3.7 years post-transplant and showed the mean FFR in the LAD was 0.88 ± 0.07 and below the normal threshold of 0.94 in 75% of cases, less than 0.80 in 15% of patients, and even less than 0.75 in 6% [16]. In the same study, IVUS was performed and invasive FFR measurements showed a strong correlation with indexes of plaque burden [16]. Hence, invasive FFR measurements seem to detect CAV before anatomical changes become apparent on ICA. In our study, similar results were found. The 28 patients in our study at 5–9 years post-HTx (and thus closest in time after HTx compared to the invasive group mentioned above) had a LAD FFRct of 0.87 (0.82–0.91). Reported invasive FFR measurements in HTx patients were performed in the distal two thirds of the LAD [6]. This likely corresponds well to the distal FFRct as vessels are segmented down to 1.8 mm. FFRct, however, enables calculation of FFR values for each location in all coronary arteries which is a major advantage and provides more comprehensive information. Yang et al. showed that patients with baseline invasive FFR of < 0.90 have a significantly lower (42% vs. 79%) event-free survival of death or retransplantation at a mean of 4.5 ± 3.5 years follow-up [6]. FFR may therefore help to identify patients at increased risk of severe morbidity and mortality and tailor medication use accordingly. In our series, the overall distal FFR was only slightly lower at longer times since HTx and the difference was not statistically significant. Three patients suffered a MACE during follow-up in our study due to iatrogenic infarct, a carcinoma, and cardiac death with unknown cause. Our follow-up period is however limited to 1 year. With longer clinical follow-up including CCTA scans, we can assess how FFRct changes over time relate to outcome. Based on data from invasive FFR measurements, there may be two different mechanisms that lead to a reduction in FFR especially in the first year after HTx [18]. Patients with negative remodeling of the coronary arteries causing reduction in vessel volume without a change in plaque volume in the first year after HTx showed a decrease in invasive FFR from 0.88 ± 0.06 to 0.84 ± 0.07 [18]. Alternatively, patients without a decrease in vessel volume but an increase in plaque volume also showed reduction in invasive FFR from 0.89 ± 0.05 to 0.85 ± 0.05 [18]. This indicates changes in vessel volume due to negative remodeling itself without plaque progression may contribute to myocardial ischemia in HTx patients. Interestingly, we found a small reduction in mean distal FFRct at longer times after HTx albeit not statistically significant. The coronary artery volume and V/M are readily available from the image segmentation to compute FFRct values and may provide valuable additional information in HTx patients regarding vessel remodeling over time [12].

Heart transplant patients with CAV may have microvascular dysfunction to some degree. In subgroups of patients prone to have microvascular dysfunction (e.g., hypertension and diabetes) FFRct accuracy is not affected [19, 20]. With microvascular disease, there may be diminished coronary flow reserve with a normal FFR. However, it does not imply that FFR is less accurate, only that there may not be epicardial disease appropriate for revascularization.

### Limitations

Given the cross-sectional nature of the study, potentially, the patients that were at a longer time after transplantation represents a group that has less severe CAV and/or slower progression of CAV.

FFRct has not been validated against invasive FFR in HTx patients. FFRct is however extensively validated in stable chest pain patients demonstrating good correlation and reproducibility of measurements [21]. A general limitation of FFRct analysis in HTx patients is that some patients cannot undergo either CCTA due to poor kidney function or FFRct analysis due to the presence of stents in two or more major coronary arteries. The follow-up time of 1 year in our study is relatively limited. Invasive FFR in heart transplant patients carries prognostic information as described by Yang et al. [6]. Longer follow-up is needed to establish if FFRct provides similar prognostic value in heart transplant patients.

### Future outlook

Anatomical coronary artery assessment on CCTA combined with additional FFRct analysis, V/M, plaque analysis, and quantification from the same CCTA dataset could provide a comprehensive non-invasive assessment in HTx patients. It will be necessary to evaluate changes over time of the above-mentioned parameters and their relation to the occurrence of adverse events during longer term follow-up. Potentially, the current CAV grading classification may even be altered and/or expanded using these parameters to more accurately identify which patients will develop CAV and how the disease will progress with the goal to determine the optimal time point to adjust medical therapy. Newer drugs like PCSK9 inhibitors have only been described in small case series without a long follow-up and at the moment, it is not known if this is a sensible adjunctive therapy in this patient category [22]. Maybe CCTA and FFRct may play a role in the evaluation of the effect of new therapies.

In conclusion, FFRct was successfully performed on CCTA scans of HTx patients and demonstrated that more than a quarter of patients had a focal coronary stenosis with FFRct ≤ 0.8. Even in the absence of a focal stenosis, FFRct values are often abnormal in HTx patients.

## Supplementary Information

Below is the link to the electronic supplementary material.

Supplementary file1 (DOCX 18 KB)

## Abbreviations

CAV: Cardiac allograft vasculopathy
CCTA: Coronary computed tomography angiography
CMV: Cytomegalovirus
CNI: Calcineurin inhibitor
FFR: Fractional flow reserve
FFRct: Coronary computed tomography angiography–derived fractional flow reserve
HTx: Heart transplant
ICA: Invasive coronary angiography
ISHLT: International society for heart and lung transplantation
IVUS: Intravascular ultrasound
LAD: Left anterior descending
LCx: Left circumflex
MACE: Major adverse cardiovascular events
mTORi: Mammalian target of rapamycin receptor inhibitor
RCA: Right coronary artery
SPECT: Single-photon emission computed tomography
V/M: Volume-to-mass ratio

## Declarations

### Guarantor

The scientific guarantor of this publication is R. Budde, MD, PHD.

### Statistics and biometry

One of the authors has significant statistical expertise.

### Methodology

• Retrospective

• cross-sectional study

• performed at one institution
