# Supplementary material for: CT-derived fractional flow reserve (FFRct) for functional coronary artery evaluation in the follow-up of patients after heart transplantation
Source: Eur Radiol. 2021 Sep 15;32(3):1843–52. doi: 10.1007/s00330-021-08246-5 (PMC8831350; doi:10.1007/s00330-021-08246-5)
Supplement: Supplementary file 1 — Supplementary file1 (DOCX 18 KB) [file 330_2021_8246_MOESM1_ESM.docx]

**Per patient comparison of FFRct, invasive coronary angiography and FFR findings.**

| Patient number | FFRct measurements | | | ICA FFR measurements and revascularization by PCI | | |
| --- | --- | --- | --- | --- | --- | --- |
|  | RCA | LAD | LCX | RCA | LAD | LCX |
| 1 | 0.64* | 0.86 | 0.89 | NP# | NP | 0.94 |
| 2 | 0.91* | 0.77* | 0.79* | NP# | NP# | NP |
| 3 | 0.98* | 0.83* | 0.87 | NP | NP# | NP |
| 4 | 0.94* | 0.50* | 0.50* | NP | NP# | NP |
| 5 | 0.88* | 0.75* | 0.91 | 0.83# $ | 0.87# $ | NP |
| 6 | 0.91* | 0.49* | 0.87 | NP | NP# | NP |
| 7 | 0.92* | 0.85* | 0.90 | 0.96 | 0.83 | NP |
| 8 | 0.50* | 0.50* | NP | 0.98 | 0.99 | NP |
| 9 | 0.94* | 0.76* | 0.71* | 0.95 | NP# | NP |
| 10 | 0.50* | 0.84* | 0.82* | NP | 0.94 | NP |
| 11 | 0.96* | 0.82* | 0.91 | NP | 0.95 | NP |
| 12 | 0.89 | 0.50* | 0.86 | NP | NP# | NP |
| 13 | 0.50* | 0.89* | NP | 0.91 | NP | NP |

FFRct and ICA FFR measurements in all patients who had an ICA after the FFRct. When a focal stenosis ≥30% was seen, the FFRct measurement of the stenosis is given and indicated by an asteriks. If no obstruction was observed, the distal value is given. In patient nr 5, additional optical coherence tomography of the RCA and LAD was performed. In patient nr 8 ICA showed chronic total occlusion of an RV branch, the distal LAD and an obtuse marginal branch but these were not treated.

FFR=fractional flow reserve; FFRct=fractional flow reserve determined on CT; LAD=left anterior descending; LCX=left circumflex artery; ICA=invasive coronary angiography; NP=FFR not performed; RCA=right coronary artery; *=FFR measurement at location of obstruction ≥30% at modelled stenoses; #=percutaneous coronary intervention performed, $=optical coherence tomography”
